# The Lysine Deacetylase RpdA Is Essential for Virulence in *Aspergillus fumigatus*

**DOI:** 10.3389/fmicb.2019.02773

**Published:** 2019-12-04

**Authors:** Ingo Bauer, Matthias Misslinger, Yana Shadkchan, Anna-Maria Dietl, Verena Petzer, Thomas Orasch, Beate Abt, Stefan Graessle, Nir Osherov, Hubertus Haas

**Affiliations:** ^1^Institute of Molecular Biology, Biocenter, Medical University of Innsbruck, Innsbruck, Austria; ^2^Department of Clinical Microbiology and Immunology, Aspergillus and Antifungal Research Laboratory, Sackler School of Medicine, Tel Aviv University, Tel Aviv-Yafo, Israel; ^3^Department of Internal Medicine II (Infectious Diseases, Immunology, Rheumatology and Pneumology), Medical University of Innsbruck, Innsbruck, Austria

**Keywords:** KDAC, RpdA, murine virulence model, *Aspergillus fumigatus*, xylose, *xylP*, conditional *in vivo* expression, HDAC

## Abstract

Current suboptimal treatment options of invasive fungal infections and emerging resistance of the corresponding pathogens urge the need for alternative therapy strategies and require the identification of novel antifungal targets. *Aspergillus fumigatus* is the most common airborne opportunistic mold pathogen causing invasive and often fatal disease. Establishing a novel *in vivo* conditional gene expression system, we demonstrate that downregulation of the class 1 lysine deacetylase (KDAC) RpdA leads to avirulence of *A. fumigatus* in a murine model for pulmonary aspergillosis. The *xylP* promoter used has previously been shown to allow xylose-induced gene expression in different molds. Here, we demonstrate for the first time that this promoter also allows *in vivo* tuning of *A. fumigatus* gene activity by supplying xylose in the drinking water of mice. In the absence of xylose, an *A. fumigatus* strain expressing *rpdA* under control of the *xylP* promoter, *rpdA*^*xylP*^, was avirulent and lung histology showed significantly less fungal growth. With xylose, however, *rpdA*^*xylP*^ displayed full virulence demonstrating that xylose was taken up by the mouse, transported to the site of fungal infection and caused *rpdA* induction *in vivo*. These results demonstrate that (i) RpdA is a promising target for novel antifungal therapies and (ii) the *xylP* expression system is a powerful new tool for *in vivo* gene silencing in *A. fumigatus*.

## Introduction

*Aspergillus fumigatus* is the most common airborne mold pathogen, capable of causing systemic disease, termed invasive pulmonary aspergillosis, mostly in immunocompromised patients (van de Veerdonk et al., 2017). Difficulties in diagnosis and the emergence of azole-resistant clinical isolates result in high mortality rates associated with invasive pulmonary aspergillosis (Meis et al., 2016; Fisher et al., 2018). Additionally, antifungal drugs used in the clinic suffer from poor specificity and side effects in patients (Campoy and Adrio, 2017). Consequently, there is an urgent need for improvement of antifungal prophylaxis, diagnosis, and therapy (Denning and Bromley, 2015).

Chromatin modulators represent potential antifungal targets. Chromatin is composed of DNA, histones and other proteins ensuring compact organization of the genetic material. The highly conserved N-terminal tails of histones are subject to a variety of post-translational modifications, which significantly impact the expression of genes. One of these modifications is the reversible acetylation of distinct lysine residues, catalyzed by lysine acetyltransferases and their counterparts, lysine deacetylases (KDACs), originally termed histone deacetylases (HDACs). The term KDAC appears more appropriate as it became evident that non-histone proteins are also subject to acetylation by the very same enzymes (reviewed by Narita et al., 2019). One example for an *A. fumigatus* non-histone protein, whose acetylation status has been proposed to have significant implications on virulence, is the heat shock protein 90 (Lamoth et al., 2014).

Most fungi have four classical KDACs (Brosch et al., 2008) belonging to two different classes. In *Aspergillus nidulans*, two class 1 enzymes (orthologs of yeast RPD3 and HOS2) and two class 2 enzymes (orthologs of yeast HDA1 and HOS3) were identified (Graessle et al., 2000). Several reports have linked KDACs to fungal virulence pathways, e.g., of the plant pathogens *Cochliobolus carbonum* (Baidyaroy et al., 2001), *Ustilago maydis* (Elías-Villalobos et al., 2015) or the human pathogens *Candida albicans* (Hnisz et al., 2009), and *Cryptococcus neoformans* (Brandão et al., 2018). Furthermore, application of KDAC inhibitors has been proposed to have an additive effect on antifungal treatment with triazoles, however, with contrasting results depending on the fungi examined (e.g., Pfaller et al., 2009; Pidroni et al., 2018).

In *A. fumigatus*, the KDAC RpdA was recently shown to be essential and was therefore proposed as a potential new target for antifungal therapies (Bauer et al., 2016). RpdA-type KDACs share a catalytic domain that is highly conserved throughout eukaryotes and that is druggable; several such inhibitors are FDA-approved for cancer treatment (West and Johnstone, 2014). Furthermore, RpdA proteins contain a conserved fungal-specific C-terminal motif required for nuclear localization, catalytic activity, and viability in axenic growth (Tribus et al., 2010; Bauer et al., 2016), which might allow development of fungal-specific KDAC inhibitors.

**FIGURE 1:**
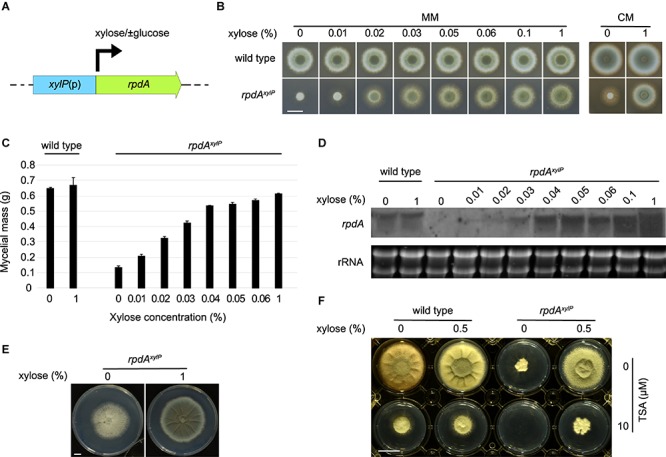
Expression of *rpdA* is tunable by xylose in strain *rpdA*^*xylP*^ during axenic growth. **(A)** Scheme of *rpdA* expression under control of the *xylP* promoter, *xylP*(p), in strain *rpdA*^*xylP*^. The arrow indicates strong induction by xylose even in the presence of glucose. The transcription start sites (34 and 38 bp upstream of the translation start) have been determined previously (Haas et al., 1993). Promoter motifs including putative binding sites for the xylose-sensing transcription factor XlnR have been predicted previously (Zadra et al., 2000). **(B)** Growth of *rpdA*^*xylP*^ and wild type on solid minimal (MM) and complex (CM) media with different xylose concentrations. Fungal strains were point inoculated (1 × 10^4^ conidia), pictures were taken after incubation for 48 h at 37°C. **(C)** Biomass measurements of *rpdA*^*xylP*^ mycelia grown in liquid minimal medium. Media were inoculated with 10^6^ conidia per ml and biomass production was determined after growth for 24 h at 37°C. Error bars represent the standard deviation of three replicates. **(D)** Northern analysis of *rpdA* expression in wild type and *rpdA*^*xylP*^ grown under different xylose concentrations. RNA was isolated after 20 h of growth in liquid minimal medium supplemented with xylose as indicated. **(E)** Growth of *rpdA*^*xylP*^ on solid MM with and without xylose. Fungal strains were point inoculated (1 × 10^4^ conidia), pictures were taken after incubation for 5 days at 37°C. **(F)** Effect of trichostatin A (TSA) on growth of wild type and *rpdA*^*xylP*^ with and without xylose induction. Fungal strains were point inoculated (1 × 10^4^ conidia) on solid MM containing 10 μM TSA solubilized in DMSO or the same DMSO concentration (0.2%) without TSA. Picture was taken after 3 days of incubation at 37°C. Scale bars represent 1 cm.

In order clarify the significance of RpdA during infection, an appropriate conditional gene expression system in a murine aspergillosis model was required. Different conditional gene expression systems have been established in *A. fumigatus* based on different promoters, e.g., the nitrate-inducible *A. fumigatus niiA* promoter, the alcohol-inducible *A. nidulans alcA* promoter, the xylose-inducible *Penicillium chrysogenum xylP* promoter (Zadra et al., 2000; Romero et al., 2003; Hu et al., 2007; Hartmann et al., 2010), and tetracycline-inducible/repressible Tet-On/Off systems exploiting the *Escherichia coli* tetracycline-resistance operon (Vogt et al., 2005). Of these, the Tet-On/Off systems have been applied for both up- and downregulation, while the *niiA* promoter was employed for downregulation of gene expression during infection (Hu et al., 2007; Lin et al., 2015; Sasse et al., 2016; Peng et al., 2018; Souza et al., 2019). The promoter of the *P. chrysogenum* β-1,4-endoxylanase-encoding *xylP* gene has been shown previously to allow xylose-mediated activation of gene expression (Figure 1A), even in the presence of glucose, during axenic growth in several molds including *A. nidulans*, *A. fumigatus*, *P. chrysogenum*, and *Penicillium marneffei* (Zadra et al., 2000; Pongsunk et al., 2005; Hartmann et al., 2010; Sigl et al., 2010; Tribus et al., 2010; Yasmin et al., 2012; Bauer et al., 2016; Vaknin et al., 2016; Gsaller et al., 2018; Misslinger et al., 2018). Moreover, xylose has been shown to be well absorbed, poorly metabolized, and quickly excreted by monogastric mammals (Huntley and Patience, 2018). These properties are already exploited in medicine to study absorption and, consequently, to assay the integrity of the gastrointestinal mucosa (Weiner et al., 1984).

Here, we demonstrate for the first time, that (i) the *xylP* promoter allows the control of *A. fumigatus* gene expression during infection in a non-neutropenic murine pulmonary aspergillosis model and (ii) downregulation of *rpdA* renders *A. fumigatus* avirulent. These data confirm RpdA as promising target for KDAC inhibitors with potential as novel agents against invasive fungal infections.

## Results

### Depletion of RpdA Leads to a Drastic but Reversible Growth Reduction Under Axenic Growth Conditions

To clarify the role of RpdA (Afu2g03390) during infection, we used for the first time the *xylP* promoter for conditional gene expression of *rpdA* in a murine aspergillosis model. Therefore, the endogenous *rpdA* promoter was replaced by the *xylP* promoter with concomitant integration of a pyrithiamine resistance cassette via homologous recombination in *A. fumigatus* AfS35, termed wild type here, resulting in *rpdA*^*xylP*^ transformants, which were verified by Southern blot analysis (Supplementary Figure S1).

Although RpdA has been shown to be essential in *A. fumigatus* by heterokaryon rescue analysis (Bauer et al., 2016), *rpdA*^*xylP*^ displayed weak growth without sporulation on solid minimal medium (Figure 1B), solid complex medium (Figure 1B) and in liquid medium without xylose-induction (Figure 1C). Xylose rescued growth of *rpdA*^*xylP*^ in a concentration-dependent manner confirming the functionality of this expression system. Interestingly, radial growth of *rpdA*^*xylP*^ did not fully reach the wild-type degree (Figure 1B), which might reflect differences in the 5′-UTR of *rpdA* in the *rpdA*^*xylP*^ strain, as the *rpdA* mRNA in *rpdA*^*xylP*^ contains the 5′-UTR of the *xylP* mRNA. Alternatively, fine-tuned regulation of *rpdA* expression during growth via the endogenous promoter might be of importance. Northern analysis confirmed downregulation of *rpdA* in the absence of xylose and a xylose-concentration-dependent induction in strain *rpdA*^*xylP*^ (Figure 1D), while xylose did not affect *rpdA* expression in the wild type.

The weak growth of *rpdA*^*xylP*^ without xylose induction might be caused by slight leakiness of the *xylP* promoter and/or by parental carry-over of xylose or RpdA via the conidia used for inoculation (see below). After 5 days of growth the colony area of *rpdA*^*xylP*^ without xylose induction reached 75% of that of *rpdA*^*xylP*^ with xylose induction (Figure 1E). An impact of parental RpdA carry-over during germination cannot be completely excluded. Nevertheless, the presented data indicate that growth of *rpdA*^*xylP*^ without xylose induction is caused by leakiness of the *xylP* promoter as dilution of parental RpdA via growth would be expected to decrease and finally block the growth *rpdA*^*xylP*^ without xylose induction within this time frame. Notably, radial growth of *rpdA*^*xylP*^ without xylose displayed significant differences to *rpdA*^*xylP*^ with xylose induction: The mycelial layer was significantly thinner (data not shown) and conidiation was largely blocked in *rpdA*^*xylP*^ without induction (Figure 1E). Similarly, RpdA was found to play a crucial role in conidiation in *A. nidulans* (Tribus et al., 2010).

The KDAC inhibitor trichostatin A (TSA, 10 μM) decreased the growth of wild type and xylose-induced *rpdA*^*xylP*^ but completely blocked growth of *rpdA*^*xylP*^ without xylose induction (Figure 1F). These data strongly indicate that RpdA is indeed the target for the growth-inhibitory activity of TSA as suggested previously in *A. nidulans* (Bauer et al., 2016).

### Xylose Feeding of Mice Leads to Rising Xylose Concentrations in Plasma

**FIGURE 2:**
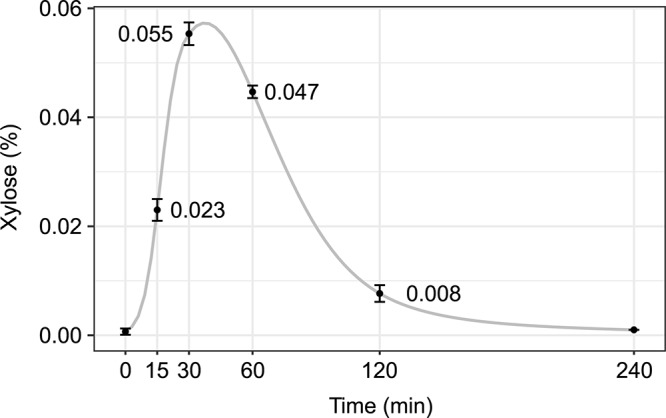
Xylose plasma levels reach concentrations that are sufficient to promote growth of *rpdA*^*xylP*^ *in vitro*. 0.2 ml of a 10% xylose solution were administered to a mouse by oral gavage. Blood samples were taken after the time points indicated and plasma xylose concentrations were measured in triplicates (mean ± standard deviation is indicated). A cubic spline interpolation line illustrating the course of xylose plasma levels is shown in gray.

To test if xylose is absorbed by mice to levels sufficient for *in vivo* induction of the *xylP* promoter, 0.2 ml of a 10% (w/v) xylose solution was administered to mice by oral gavage, followed by determination of the plasma xylose concentration 15–240 min after xylose ingestion (Figure 2). No xylose was detected in plasma samples taken directly after the gavage, indicating the absence of xylose under conventional nutritional conditions. Already after 30 min, a plasma xylose concentration of 0.055% was reached. The xylose concentration decreased after 60 min and was hardly detectable after 120 min indicating efficient clearing. As reported previously, mice drink 4–8 ml water within 24 h (Bachmanov et al., 2002), resulting in approx. 0.25 ml/h calculated water uptake (Li et al., 2016). These data indicated that drinking of xylose results in sufficient concentrations for *in vivo* induction of the *xylP* promoter.

### Downregulation of *rpdA* Causes Avirulence

Next, the *rpdA*^*xylP*^ strain was tested for virulence in a non-neutropenic pulmonary infection model, in which mice are immunosuppressed with cortisone acetate. Three days after the first immunosuppression, cohorts of ten mice were infected intranasally with wild-type or *rpdA*^*xylP*^ conidia and were supplied with drinking water with or without xylose (10% m/v). For mice infected with the wild-type strain, the xylose diet did not affect mortality (Supplementary Figure S2), weight loss, or drinking behavior (data not shown). The latter is interesting as xylose supplementation results in a sweet taste of the drinking water, which might affect drinking behavior.

**FIGURE 3:**
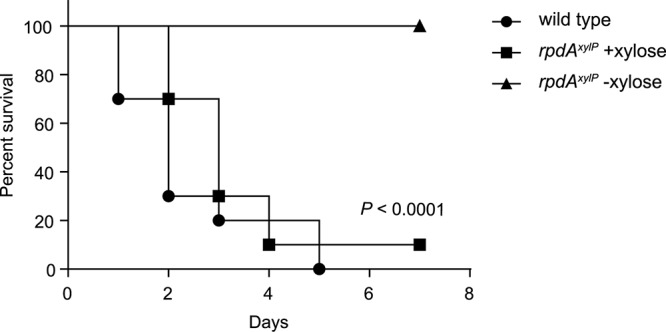
Downregulation of *rpdA* causes avirulence. Mouse survival curves (Kaplan–Meier plot) following intranasal infection of cortisone-acetate immunocompromised mice (*n* = 10 animals/group) with (+) and without (–) 10% xylose in the drinking water. Comparison of survival curves for *rpdA*^*xylP*^−xylose vs. wild type or *rpdA^*xylP*^* +xylose using the log-rank test (Mantel-Cox) revealed significance at *P* < 0.0001.

**FIGURE 4:**
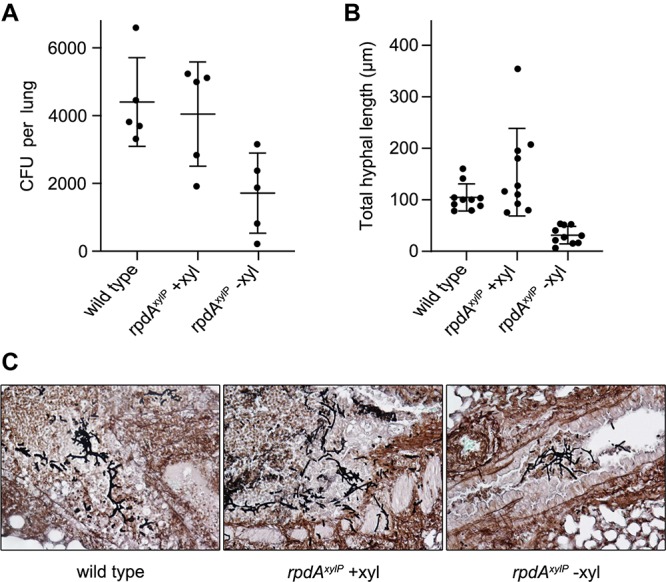
Histology reveals reduced fungal colonization by avirulent *rpdA*^*xylP*^ in the absence of xylose. Lungs were extracted 2 days post intranasal infection of cortisone-acetate immunocompromised mice. **(A)** Mouse lung fungal burden is reduced in mice infected with *rpdA*^*xylP*^−xylose (xyl) compared to wild type (*P* = 0.0094) and *rpdA^*xylP*^* +xylose (*P* = 0.0292). **(B)** Hyphal lesions from *rpdA*^*xylP*^ –xylose contain significantly less fungal hyphae (*P* < 0.0001) than those from *rpdA^*xylP*^* +xylose and wild type. The extend of fungal growth was measured by manually tracing the combined length of hyphae in each lesion (*n* = 10) using ImageJ software. **(C)** Representative lung sections of infected mice. Staining, performed with gomori methenamine silver (GMS), stains fungi black.

The *rpdA*^*xylP*^ strain with xylose supplementation showed mortality rates similar to that of the wild-type strain without xylose in the drinking water (Figure 3). In contrast, *rpdA*^*xylP*^ without xylose was avirulent (*P* < 0.0001, Figure 3). Histological analysis 2 days post infection revealed a similar progress in germination of wild-type and *rpdA*^*xylP*^ strains with and without xylose (Figure 4C), which is in agreement with the growth of *rpdA*^*xylP*^ under both inducing and non-inducing axenic conditions (Figures 1B,C), although the growth was very limited under non-inducing conditions. The *rpdA*^*xylP*^ mutant without xylose caused a lower lung fungal burden compared to *rpdA*^*xylP*^ with xylose (*P* = 0.0292, Figure 4A) or wild type without xylose (*P* = 0.0094, Figure 4A). Moreover, examination of histological lung sections revealed less and smaller fungal lesions in mice infected with *rpdA*^*xylP*^ maintained without xylose, as determined by measuring the total length of fungal hyphae within 10 infection foci each of *rpdA*^*xylP*^ with and without xylose and wild-type (*P* < 0.0001, Figures 4B,C). These results match the partial growth of *rpdA*^*xylP*^ in the absence of xylose during axenic growth conditions (Figures 1B,C).

Taken together, these data demonstrate that downregulation of *rpdA* (*rpdA*^*xylP*^ under non-inducing conditions), which allows limited axenic growth (Figures 1B,C), permits germination during infection (Figures 4B,C) but is insufficient to support virulence (Figure 3). Moreover, the avirulence of *rpdA*^*xylP*^ without xylose supplementation compared to the virulence of *rpdA*^*xylP*^ with xylose supplementation (Figure 3) demonstrates that the *xylP* promoter allows control of expression during infection by xylose feeding.

## Discussion

It has been shown that the class 1 KDAC RpdA is essential for growth of both *A. nidulans* and *A. fumigatus* under axenic conditions (Tribus et al., 2010; Bauer et al., 2016). Concordantly, pharmacological KDAC inhibition resulted in strong growth inhibition (Bauer et al., 2016). Here, we employed for the first time the *xylP* promoter, which has previously been shown to allow xylose-mediated induction of gene expression in several mold species during axenic conditions, for modulation of *A. fumigatus* gene expression during murine infection.

The observed wild-type-like virulence of *rpdA*^*xylP*^ under xylose supplementation demonstrates that the *xylP* inducer xylose indeed reaches the infected tissue in amounts sufficient to induce *rpdA* to a level that can rescue virulence. The employed *xylP* system therefore appears to be a suitable new tool to conduct virulence studies as an interesting alternative to gene deletion mutants or expression systems that are induced or repressed by tetracycline/doxycycline (Tet-On/Tet-Off promoter systems). The latter systems have been introduced for *A. fumigatus* in several axenic approaches (Vogt et al., 2005; Helmschrott et al., 2013; Kalb et al., 2015; Macheleidt et al., 2015; Wanka et al., 2016) and there are four studies employing these promoters for control of *A. fumigatus* gene expression during infection: three for Tet-On (Lin et al., 2015; Sasse et al., 2016; Souza et al., 2019) and one for the Tet-Off system (Peng et al., 2018). A disadvantage of administration of tetracycline-type antibiotics might be their potential side effects in host mitochondria and on the animal microbiome (Ahler et al., 2013; Chatzispyrou et al., 2015; Moullan et al., 2015). Moreover, the Tet-On/Tet-Off systems require not only exchange of the promoter, as in the *xylP* system, but additionally incorporation of the Tet transcription factor (Lin et al., 2015; Sasse et al., 2016). The half-life of doxycycline in blood was determined to be approximately 170 min in healthy mice (Böcker et al., 1981, 1984). Due to the shorter *in vivo* half-life of xylose in blood of approximately 35 min, the *xylP* promoter system might be an attractive alternative, providing different kinetics.

As RpdA has been shown previously to be essential for growth (Bauer et al., 2016), the limited growth of the *rpdA*^*xylP*^ strain in the absence of xylose indicates slight leakiness of the *xylP* promoter. Expression of various genes under control of the *xylP* promoter in several mold fungi caused a loss-of-function phenotype under non-inducing conditions (Pongsunk et al., 2005; Hartmann et al., 2010; Sigl et al., 2010; Fazius et al., 2012; Gsaller et al., 2012; Bugeja et al., 2013; Baldin et al., 2015; Vaknin et al., 2016), e.g., growth of mutant strains expressing essential genes of the iron sulfur cluster biosynthetic machinery under control of the *xylP* promoter was completely blocked in non-inducing conditions in *A. fumigatus* (Misslinger et al., 2018). In contrast, use of the *xylP* promoter for expression of the essential monothiol glutaredoxin encoding *grxD* gene in *A. fumigatus* indicated leakiness of the *xylP* promoter dependent on the *grxD* allele (Misslinger et al., 2019). Most likely, the consequence of promoter leakiness, as monitored by growth pattern, depends on the gene expressed; i.e., it is affected by the required amount of the gene product, whereby the efficiency of expression is influenced by mRNA and protein stability, which are gene-specific features. Moreover, the genomic integration site might affect promoter leakiness. Similar to *A. fumigatus*, *xylP* promoter-driven *rpdA* without induction allowed minimal growth also in *A. nidulans* (Tribus et al., 2010).

At first glance, promoter leakiness might appear unfavorable. Under certain circumstances, however, it actually might be advantageous. The avirulence of *rpdA*^*xylP*^ without xylose induction underlines that even a decrease and not only absence of *rpdA* expression is sufficient to impair pathogenicity. This result actually emphasizes the importance of RpdA as a virulence factor. Moreover, residual RpdA activity in *rpdA*^*xylP*^ strains under repression mimics a realistic drug treatment scenario much more than an entire blocking of gene expression, as drug-mediated therapies hardly ever cause total inhibition of the respective targets. Consequently, only few therapy regimens achieve the effect of a total inactivation of the corresponding targets or physiological pathways. Furthermore, in studies evaluating potential drug targets with molds, generally conidia are used for pathogen inoculation. Very often it is observed that the avirulence of mutants is based on the lack of germination of the conidia in the lung. However, this does not reflect the real scenario for therapeutic intervention, as treatment of patients has to combat hyphae but not conidia. In this respect, *xylP* promoter-mediated downregulation of gene expression represents an interesting alternative to gene deletion, not only for the evaluation of the role in virulence of essential, but also for non-essential genes. On the other hand, regarding a potential broader application, promoter leakiness could indeed impede significant knock-down, particularly of weakly expressed genes.

Growth of *rpdA*^*xylP*^ without xylose induction was completely blocked by the KDAC inhibitor TSA in a concentration that caused only partial growth inhibition of wild type or xylose-induced *rpdA*^*xylP*^. These data indicate that the antifungal effect of TSA is mainly due to inhibition of catalytic RpdA activity.

The avirulence of the *rpdA*^*xylP*^ strain under non-inducing conditions might be attributed to its reduced growth. Remarkably, deletion of the mitogen-activated protein kinase MpkA-encoding gene caused a dramatic growth defect without diminishing virulence (Valiante et al., 2008). The latter indicates that growth rate and virulence are not always strictly linked. If avirulence of *rpdA*^*xylP*^ under non-inducing conditions is caused by the growth defect or a deregulation of virulence-relevant pathways remains to be elucidated.

Irrespective of the mode of action of RpdA, several KDAC inhibitors have already been clinically approved by the FDA for therapeutical use against certain types of cancer: e.g., vorinostat, panobinostat, belinostat, and romidepsin (West and Johnstone, 2014). It is, however, not clear yet, to which extent these compounds inhibit RpdA activity or affect growth of *A. fumigatus in vitro* or *in vivo*. Importantly, RpdA itself seems to be a promising candidate for the development of fungal-specific KDAC inhibitors due to specific structure variations within its catalytic domain and the presence of a conserved fungal-specific C-terminal motif (Bauer et al., 2016).

Taken together, in this study we demonstrate the crucial role of RpdA in virulence of *A. fumigatus* employing a novel expression system, which enables tuning of expression during the infection process.

## Materials and Methods

### Strains and Media

For this study, the *rpdA* promoter was exchanged by the *xylP* promoter in the strain AfS35, which lacks non-homologous end joining due to deletion of the *akuA* gene (Krappmann et al., 2006). Information on these strains is given in Table 1. Fungal strains were propagated in *Aspergillus* minimal medium (AMM: 1% (m/v) glucose, 20 mM glutamine, salt solution and trace elements according to Pontecorvo et al., 1953) and in *Aspergillus* complete medium (CM: 2% (m/v) glucose, 0.2% (m/v) peptone, 0.1% (m/v) yeast extract, 0.1% (m/v) casamino acids, salt solution and trace elements). Addition of xylose is indicated separately.

**TABLE 1:** Strains used in this study.

**Name**	**Genotype**	**Background/FGSC#**	**References**
AfS35 (wild type)	*akuAΔ:loxP*	D141/A1159	Krappmann et al., 2006
TIB104 (*rpdA*^*xylP*^)	*rpdA(p):ptrA, xylP(p):rpdA; akuAΔ:loxP*	AfS35	This study

### Gene Cassette and Strain Generation

For generation of the *rpdA*^*xylP*^ allele, protoplast fusion (Tilburn et al., 1983) combined with the split-marker technique (Nielsen et al., 2006) was used. *A. fumigatus* AfS35 was co-transformed with 2 DNA fragments, each containing 0.5-kb overlapping but incomplete fragments of the pyrithiamine resistance-conferring *ptrA* allele from *Aspergillus oryzae* (Kubodera et al., 2000) ligated to 1.2- and 1-kb of *A. fumigatus rpdA* 5′-UTR and *rpdA* coding regions, respectively. These fragments were amplified from plasmid pIB24, which was constructed as follows: First, the *rpdA* 5′-UTR was amplified using primers *AfrpdA*5UTRfwd*Kpn*I (AAATGGTACCATCCAACGACTGTTTACCATCCAACGACT GTT) and *AfrpdA*5UTRrev*Nde*I (TTTATAATACATATGTGAG AAAGGTCGTGTATGTGAGAAAGGTCGTG). The resulting product was digested with *Kpn*I and *Nde*I and cloned into pSK275 (Szewczyk and Krappmann, 2010) harboring the pyrithiamine resistance cassette digested with the same enzymes, yielding pSK275-*rpdA*5′UTR. Next, a fragment containing the *P. chrysogenum xylP* promoter (Zadra et al., 2000) fused to the *rpdA* coding sequence was inserted at the *Sma*I site of pSK275-*rpdA*5′UTR and screened for proper orientation by restriction analysis. To this end, the *xylP* promoter fragment was amplified using primers *xylP*f2 (CCCGGGCACTGATGCGAGC AACAG) and *AfrpdAxylP*r (CGGAGGAGCAGCCATGTT GGTTCTTCGAGTCG) with p*xylPrpdA*TRU0 (Tribus et al., 2010) as template and then spliced by overlap extension (Shevchuk et al., 2004) to a part of the coding sequence of *rpdA* that was amplified from *A. fumigatus* genomic DNA with primers *xylPAfrpdA*f (CGACTCGAAGAACCAACATGGCTGCTCCTC CGATTG) and *AfrpdA*7r (ACTGTCACCACCACACTG). All PCR reactions were performed using the *Pfu*-X7 polymerase (Nørholm, 2010).

### RNA Isolation and Northern Blot Analysis

RNA was isolated with TRI Reagent (Sigma) according to the manufacturer’s manual. 10 μg of RNA was used for electrophoresis on a 0.6 M formaldehyde agarose gel and blotted on Hybond-N-membranes (Amersham). A digoxigenin (DIG)-labeled *rpdA* probe was amplified by PCR using primers *AfrpdA*5f (CAAGTACGGAGAATACTTCC) and *AfrpdA*6r (CTTCGTCAAGGTCATCCAG) and detected with an anti-DIG-AP conjugate and the chemiluminescent substrate CSPD (Sigma).

### Southern Blot Analysis

Genomic DNA was extracted by PCI and precipitated with isopropanol. For verification of correct *xylP* promoter integration (*xylP*(p)*:rpdA*), DNA was digested with *Pst*I and signals were detected with DIG-labeled probes against the 5′ part of the *rpdA* coding sequence. Correct integration at the *rpdA* locus yielded a signal at 3422 bp. The DIG-labeled *rpdA* probe was amplified from pIB24 by PCR using oligonucleotides *xylP*f3 (TTCATCGACTCGAAGAAC) and *AfrpdA*r2 (GTCGCATTTGTTGCGGTTAAG).

### Plasma Xylose Measurements

Mouse plasma xylose concentrations were determined spectrophotometrically using the commercially available *D*-Xylose-Assay-Kit (Megazyme, Ireland) according to the manufacturer’s instructions.

### Virulence Studies

Six-week-old female ICR mice were immunocompromised by subcutaneous injection with cortisone acetate (300 mg/kg) 3 days prior to infection, on the day of infection, and 3 and 7 days after infection. Conidia were collected in PBS with 0.2% Tween 20. Mice were infected intranasally with 5 × 10^5^ dormant conidia, suspended in 20 μl of PBS with 0.2% Tween 20 (10 μl in each nostril). Mortality was monitored for 21 days. The statistical differences for mouse survival were calculated using the log-rank (Mantel-Cox) test. Differences were considered significant at *P-*values ≤0.05. For histology and lung fungal burden estimation, 5 mice/group were treated and infected as described above and sacrificed after 48 h for lung extraction. For histology, lungs were paraffin-embedded, sectioned (3–4 μm) and stained with Gomori methenamine silver. Histology section preparations were observed using a BX51 microscope (Olympus, Milan, Italy), and images were captured using a high-resolution DP71 camera (Olympus). Lung fungal burden was assessed by homogenizing the lungs and plating dilutions on Sabouraud dextrose agar (Difco) +0.5% xylose plates for CFU (colony forming unit) enumeration. This study was carried out in accordance with the recommendations of the Ministry of Health (MOH) Animal Welfare Committee, Israel. The protocol was approved by the MOH Animal Welfare Committee, Israel.

## Data Availability Statement

All datasets generated for this study are included in the article/Supplementary Material.

## Ethics Statement

The animal study was reviewed and approved by Ministry of Health (MOH) Animal Welfare Committee, Israel.

## Author Contributions

IB, MM, YS, A-MD, VP, TO, and BA generated the data. IB, MM, SG, NO, and HH conceived and designed the experiments, and wrote the manuscript. IB, MM, YS, SG, NO, and HH analyzed the data.

## Conflict of Interest

The authors declare that the research was conducted in the absence of any commercial or financial relationships that could be construed as a potential conflict of interest.
